# Methylene Blue in the Treatment of Cancer

**Published:** 1907-05

**Authors:** Henry R. Slack

**Affiliations:** President of Georgia Pasteur Institute, Atlanta, Ga.; Superintendent LaGrange Sanatorium, LaGrange, Ga.


### METHYLENE BLUE IN THE TREATMENT OF CANCER.*

HENRY R. SLACK, PhM., M.D.,
President of Georgia Pasteur Institute, Atlanta, Ga.; Superintendent, LaGrange Sanatorium, LaGrange, Ga.

Cancer has the whole world for its field and destroys all sorts and conditions of men. While more common among the old, the young are not entirely exempt, as Osler reports six cases of cancer of the stomach in the first three decades of life.

The wise old father of medicine, Hippocrates, writing about 400 B. C., has this to say of the treatment of cancer: "The deep-seated forms are best untreated, for if treated the patient soon dies; otherwise, he might hold on for a long time." Inaccessible cancer is two and a half times more frequent than accessible; therefore, any treatment that gives reasonable assurance of prolonging life and not shortening it, as was the case in the days of Hippocrates, is welcomed by the physician and is a boon to suffering humanity. Especially is this true when the administration is as simple as the one that I will present to-day.

Ten years since, I read a paper before this Association entitled, "Blue Pyoktanin in the Treatment of Inoperative Malignant Growths." This paper was published in the Journal of the American Medical Association, June 27th, 1897, and was pretty widely commented on. Shortly after this, the X-Ray began to be applied in the treatment of cancer with such success that all other methods, except surgical, were completely eclipsed by this new and wonderful light that possessed such marked therapeutic power. The novelty of roentgenization had not worn off before the discovery of that new element, radium, added a still more mysterious weapon to the therapeutic aramentarium. The extravagant claims of the users from the radiations of this new element, have not been borne out by the

*Read before the Medical Association of Georgia, in Savannah, April 17th-19th, 1907.

experience of careful clinicians, and we are again turning our attention to some of the older methods. Even Dr. Willy Meyer, of New York, who first introduced blue pyoktanin in the treatment of cancer in America, seemed to have been carried away by the new agents; but not so that sturdy old nestor in therapeutics, Dr. Abraham Jacobi. He has kept quietly using in his immense practice methylthionin hydrochlorid, as it is now called, or methylene blue, in the treatment of inoperable malignant growths, until he has now treated 150 cases, as reported at the last meeting of the American Medical Association in Boston.

Herbert Spencer says that our judgment is based on our personal experience. My personal experience with methylene blue has been very satisfactory, though I have had to abandon the hope, expressed in my paper ten years ago, of effecting a cure by its use alone. Still, the benefits derived are so pronounced that I feel justified in continuing its administration in such cases.

I have found the treatment then used quite painful, so have abandoned the technique suggested by Von Mosetig and Willy Meyer of parenchymatous injection of a two per cent. aqueous solution, for Jacobi's of simple administration by mouth. I give a tablet containing methylene blue, 2 grs., extract of belladonna, 1/4 gr. each, after each meal. If there is an ulcerating surface accessible, I dust with a four per cent. or 5 per cent. powder, or use a two per cent. injection daily. I also use the X-Ray if on the surface breast or face, as I have seen a number of cures follow the careful, judicious application of roentgenization.

I reported five cases in my former paper and have since treated twenty-nine, but will only give three, as they are fairly representative.

Case 17, Mrs. M. E. B,, age 56, widow, had nine children and three miscarriages. Complains of large and painful lump in right breast and under arm. Family history, negative; personal history, has had usual diseases of childhood, menstruation, fifteen years, regular, passed the climacteric period at forty-eighth year without incident. Has had typhoid fever, pneumonia, and malaria. Health not good for last seven years. Present trouble started in October, 1899, with a lump in breast, but did not give much pain or grow rapidly until January, 1903. Appetite good and bowels regular.

Examination: Fairly well nourished woman, heart and lungs negative; has tumor in right breast as large as goose egg; adherent and nipple retracted; axillary glands indurated. Case went to St. Joseph's Atlanta, for operation, but surgeon said was inoperable carcinoma.

Treated with X-Ray for ten weeks. The indurations in axilla entirely disappeared and the tumor in breast reduced to size of chestnut. She then left me and I did not see her until February 1st, 1904. She had been treated by a local physician with X-Ray and had bad burn, which had to be healed before treatment could be resumed. After six weeks, she was in fine condition.

October 25th, 1906, she returned very feeble and oachectic, with a foul ulcer above the nipple over an inch in diameter and also two ulcers in the axilla. The arm was very painful, much swollen and could not be used at all. This time we used the methylene blue tablets internally and applied the powder to the ulcers daily, as well as X.-Ray it occasionally. Her improvement was marked, and she is now free from pain and doing nicely, though not cured.

Case 21, J. W. M., age 46, male, farmer. Complains of sore on lower lid of left eye. Family history: Father had a similar place. Personal History: Had usual diseases of childhood, denies venerial disease, general health good. Present trouble started with a crust and scab over two years ago, and has gradually grown larger until now it is larger than a dime. Appetite sound and bowels constipated.

Examination: Well-nourished man, heart and lungs negative; has epithelioma size of dime under left eye. Treated with X-Ray three times a week for six weeks, and ulcer reduced in size to a grain of wheat and gave no trouble, but never did heal or disappear until used methylene blue internally and applied the powder to surface. Has now been well several months.

Case 26, L. J. N., age 46, colored, female, married, one child. First consulted me thirteen years ago for dysmenorrhœa and sterility. Found stenosed cervix. Dilated, and ten months afterward she gave birth to a fine girl. Did not see her again until nearly five years later. Was then complaining of pain in back, leucorrhœa, etc. Examination showed lacerated cervix with ulceration.

January, 1907, her husband said she was "Mighty bad off, sick in bed, suffers all the time and bleeds a great deal."

Dr. McCall, my assistant, called to see her and found exfoliating carcinoma of uterus, involving the vaginal wall, and inoperable. He gave methylene blue and an injection of a two per cent. solution daily. She is now up, free from pain, and able to do her house-work.

I have selected these three cases not because they have given the most brilliant results, but for their peculiar interest.

Case 17 had been pronounced inoperable carcinoma of breast over four years ago, had yielded for a time to X-Rays, but after several burns tolerance was established and the disease spread, causing the methylene blue, the disease again yielded, and the patient is ing the Methylene Blue, the disease again yielded, and the patient is now enjoying fairly good health and is free from pain.

Case 21 was an epithelioma of lower lid of eye, which was much benefited by X-Rays, but did not get well until used the Methylene Blue.

Case 26, carcinoma of uterus following laceration of cervix, which is being arrested by administration of methylene blue alone.

Of the twenty-nine cases treated with X-Ray or combined with methylthionin hydrochlorid, twelve have been dismissed as cured and five have gone over three years without recurrence. The other seven, ranking from five to thirty months since dismissal, show no evidence of return. There are nine still under observation, seven of whom are doing fairly well. As the disease is in abeyance, they suffer very little and are able to attend to their usual duties.

Of the twelve cases cured, eight were epitheliomas, three carcinomas, and one was sarcoma.

My experience with the methylthion hydrochlorid teaches me to expect the following results: 1st. Every thing with which it comes in contact is stained a deep blue; the urine soon looks like blue ink, and you should always tell your patient to expect this. 2nd. There is surcease from pain. 3rd. The general health of the patient is improved and years are added to his life.

It thus helps us to carry out the noble aim of our profession,—"To alleviatesuffering and prolong life."

Literature: Journal of American Medical Association, June 27, 1897; Journal of American Medical Association, June 9, 1906; Journal of American Medical Association, November 10, 1906; Osler's Practice of Medicine, Sixth Edition.

